# CYP2D6 intermediate metabolizer status could slow down *Plasmodium falciparum* gametocyte clearance after single-dose primaquine

**DOI:** 10.1186/1475-2875-13-S1-P69

**Published:** 2014-09-22

**Authors:** Helmi Pett, Chris Drakeley, Chi Eziefula, Mikko Neuvonen, Kjerstin Lanke, Robert Sauerwein, Mikko Niemi, Teun Bousema

**Affiliations:** 1 Radboud University Medical Center, Nijmegen, The Netherlands; 2 London School of Hygiene & Tropical Medicine, London, UK; 3 University of Helsinki and Helsinki University Central Hospital, Helsinki, Finland

## Background

The use of single-dose primaquine to block transmission of *Plasmodium falciparum* malaria is complicated by variation in glucose-6-phosphate-dehydrogenase (G6PD) activity and potentially cytochrome P450 2D6 (CYP2D6). G6PD deficiency determines primaquine safety while the CYP2D6 metabolizer phenotype may determine primaquine efficacy by influencing metabolism into active metabolite(s). While there is evidence that the CYP2D6 metabolizer phenotype influences primaquine efficacy against *P. vivax* hypnozoites, there is currently no evidence for CYP2D6 in relation to its gametocytocidal activity in *P. falciparum*.

## Materials and methods

We used samples from a dose-finding study where Ugandan children with uncomplicated malaria were randomized to receive artemether-lumefantrine together with a placebo or 0.1, 0.4 or 0.75 mg/kg primaquine. Gametocyte carriage was determined by Pfs25 QT-NASBA on nucleic acids from 50 μL blood samples that were subsequently used for CYP2D6 assays. Using the QuantStudio 12K Flex system and 32SNP OpenArray as well as copy number variation (CNV) assays, CYP2D6 genotypes were determined for the available DNA samples.

## Results

247 out of 468 individuals included in the trial were successfully genotyped for CYP2D6. CYP2D6 phenotypes were predicted from the genotypes with the classical method, yielding phenotypes for all but 13% of genotyped individuals. The number of poor metabolizers (PM) and ultrarapid metabolizers (UM) in this Ugandan cohort was very low (2% for both), while 25% (62/247) of the children were intermediate metabolizers (IM). Gametocyte prevalence on day 7 after initiation of treatment with 0.4 mg/kg primaquine was 7% (2/28) in extensive metabolizers (EM) or UM compared to 38% (3/8) in IM and 100% (1/1) in PM (*P* = 0.009). The same pattern was not present in the other treatment arms.

## Conclusions

The number of PM is low in our study population and does not allow a firm conclusion on its impact on gametocyte clearance following primaquine. IM had longer persisting gametocytes after receiving 0.4mg/kg primaquine suggesting that the transmission-blocking effect of low-dose primaquine may be influenced by CYP2D6 activity. Larger studies that include mosquito infectivity outcomes are needed to firmly establish whether CYP2D6 variations impact the utility of primaquine for *P. falciparum* transmission reducing activities.

